# The Neurovascular Unit in Glaucomatous Neurodegeneration

**DOI:** 10.3389/fcell.2020.00452

**Published:** 2020-06-16

**Authors:** Lauren K. Wareham, David J. Calkins

**Affiliations:** Department of Ophthalmology and Visual Sciences, Vanderbilt Eye Institute, Vanderbilt University Medical Center, Nashville, TN, United States

**Keywords:** neurodegeneration, glaucoma, neurovascular unit, vasculature, neurovascular coupling, gap junctions

## Abstract

Glaucoma is a neurodegenerative disease of the visual system and leading cause of blindness worldwide. The disease is associated with sensitivity to intraocular pressure (IOP), which over a large range of magnitudes stresses retinal ganglion cell (RGC) axons as they pass through the optic nerve head in forming the optic projection to the brain. Despite clinical efforts to lower IOP, which is the only modifiable risk factor for glaucoma, RGC degeneration and ensuing loss of vision often persist. A major contributor to failure of hypotensive regimens is the multifactorial nature of how IOP-dependent stress influences RGC physiology and structure. This stress is conveyed to the RGC axon through interactions with structural, glial, and vascular components in the nerve head and retina. These interactions promote pro-degenerative pathways involving biomechanical, metabolic, oxidative, inflammatory, immunological and vascular challenges to the microenvironment of the ganglion cell and its axon. Here, we focus on the contribution of vascular dysfunction and breakdown of neurovascular coupling in glaucoma. The vascular networks of the retina and optic nerve head have evolved complex mechanisms that help to maintain a continuous blood flow and supply of metabolites despite fluctuations in ocular perfusion pressure. In healthy tissue, autoregulation and neurovascular coupling enable blood flow to stay tightly controlled. In glaucoma patients evidence suggests these pathways are dysfunctional, thus highlighting a potential role for pathways involved in vascular dysfunction in progression and as targets for novel therapeutic intervention.

## Introduction

Glaucoma is an age-related disease of the visual system and a leading cause of irreversible blindness worldwide (WHO, 2020). Clinical classification schemes of the several forms of glaucoma hinge upon a key anatomic feature of the anterior segment, the iridocorneal angle, which is defined by the angle formed where the iris and cornea meet. In open-angle glaucoma, the angle is sufficiently wide to allow normal outflow of aqueous humor from the anterior chamber to the drainage canals in the trabecular meshwork at the base of the cornea. In the most common form of the disease, primary open-angle glaucoma (POAG), the angle is open but there is progressive resistance within the outflow pathways that can lead to an increase in intraocular pressure (IOP). The disease causes degeneration of the optic nerve through sensitivity to IOP which remains the only modifiable risk factor. Over a range of magnitudes, IOP stresses retinal ganglion cell (RGC) axons as they pass unmyelinated through the optic nerve head (ONH) and form the myelinated segment of the nerve and visual projection to the brain. In the anterograde direction from the ONH, axon degeneration involves transport dysfunction and eventual disassembly with subsequent pruning of synaptic termination sites in central projection sites in the brain (Calkins, 2012). In the retrograde direction back toward the retina, RGC dendritic arbors shrink and lose complexity as excitatory synapses are eliminated, though the cell body and unmyelinated axon segment persist until later in progression (Buckingham et al., 2008; Calkins, 2012). In late stages of disease progression, RGCs degenerate completely and retinal nerve fiber layer (RNFL) thickness is significantly decreased.

That the ONH is a critical juncture for pathogenic processes that underlie neurodegeneration in glaucoma is underscored by its unique structure and physiology (Sigal and Ethier, 2009; Burgoyne, 2011; Tamm et al., 2017; Lawlor et al., 2018). Through its architecture, complex IOP-dependent forces at the ONH translate to biomechanical stress at the lamina cribrosa and ultimately, to RGC axons as they pass through (Yan et al., 1994; Burgoyne et al., 2005; Downs, 2015). The ONH is also an important site for both systemic and local vascular dysfunction that likely contributes to progression. Glaucoma involves significant comorbidity with multiple vascular conditions, including migraine, arterial hypertension and hypotension, low ophthalmic artery blood pressure, and diabetes mellitus (Dienstbier et al., 1950; Hayreh, 1969, 2001). Vascular dysfunction and insufficiency at the ONH as well as in the retina can lead to ischemia that contributes to RGC degeneration (Hayreh et al., 1970; Flammer, 1984). In normal tension glaucoma, which occurs without overt elevations in IOP, vascular dysfunction may be a primary driver of disease progression through increased oxidative stress at the level of the retina and ONH (Trivli et al., 2019). Mild and repetitive hypoxic events due to small fluctuations in IOP may lead to an unstable oxygen supply, generating chronic, low-grade ischemia-reperfusion injury that differs from sustained hypoxic insults resulting from acute elevations in IOP (Flammer, 2001; Nita and Grzybowski, 2016). In both cases, however, the main consequence is progressive oxidative stress that challenges the metabolic resources RGCs require in transmitting the retinal image to the brain.

Despite the association between systemic vascular dysfunction and glaucoma, controversy remains concerning the extent of involvement of neurovascular dysfunction in RGC degeneration during glaucoma (Hayreh, 2001). Much of the data addressing vascular changes in eyes of patients have been collected using techniques that have limitations; for example, limitations in the technology available to accurately measure blood flow in the retina and ONH (discussed below). Difficulties arise when discerning whether vascular abnormalities precede glaucomatous degeneration as most studies of the vasculature in patients are carried out in those already clinically diagnosed with glaucoma (for a recent review see Ahmad, 2016). Nevertheless, there is mounting evidence to support a role for vascular dysfunction in some cases of glaucoma. Critical questions still remain, including (1) whether vascular changes precede other insults thereby increasing RGC susceptibility, (2) if vascular dysfunction follows neuronal degeneration from a breakdown in neurovascular coupling, and (3) whether particular vascular pathways are dysfunctional and, if so, if they are targets for therapeutic intervention. In the sections that follow, we will review known facts that address these questions and others that have bearing on the vascular contribution to glaucomatous neurodegeneration.

## Vascular Dysfunction in Glaucoma

**FIGURE 1:**
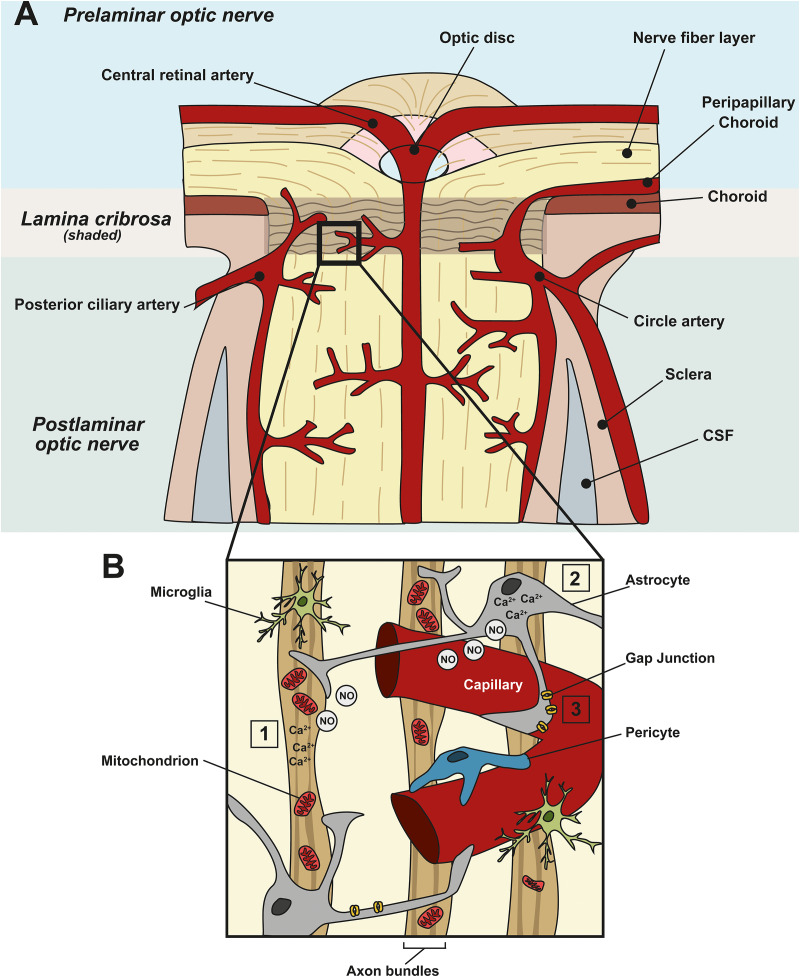
Neurovascular coupling in the ONH and vascular dysfunction in glaucoma. **(A)** Schematic showing the blood supply at the ONH. Prelaminar, lamina cribrosa, and postlaminar sections are indicated. The primary blood supply to the ONH and retina comes from the choroid, the central retinal artery, the posterior ciliary artery and the circle artery. **(B)** Enlargement of the boxed area in **(A)** showing a capillary and its associated cells in the ONH. Astrocytes, pericytes, and endothelial cells of blood vessels constitute the neurovascular unit (NVU), to link local neuronal activity to vascular changes. In healthy tissue, when there is a spike in neuronal activity (1), or metabolic demand, it leads to an increased intracellular concentration of Ca^2+^ in neurons and (2) astrocytes. This, in turn, leads to the generation of nitric oxide (NO), a vasoactive gaseous messenger, which can diffuse to nearby blood vessels, altering blood flow. In glaucoma, apoptotic neurons and reactive astrocytes lead to the breakdown of this coupling. (3) In addition, in glaucoma, ischemia, and perfusion instability damages astrocyte–astrocyte gap junctions, leading to miscommunication between astrocytes and neurons.

The high metabolic nature of the retina necessitates a continual supply of metabolites and removal of oxidative waste (Buttery et al., 1991; Wong-Riley, 2010; Country, 2017). The retina has a conflicting requirement of blood supply and minimal interference with light. The evolution of two vascular supplies meet this conflict: the choroid supplies photoreceptors that comprise one-third of the retina, and intra-retinal vessels supply the remaining two-thirds of the retina (Kur et al., 2012). The inner layers of the retina that require a proximal blood supply include the outer plexiform layer, the inner plexiform, and the ganglion cell layer (Dowling, 1987). The vascular system supplying the ONH is more complex than in the retina (Harris et al., 2005). Blood flow to the ONH is primarily supplied by the posterior capillary artery circulation via the peripapillary choroid and short posterior ciliary arteries, except for the surface nerve fiber layer which is supplied from the central retinal artery circulation (Onda et al., 1995) (Figure 1A). Blood flow regulation at these sites involves multiple metabolic and vasoactive pathways (Figure 1B).

In all stages of glaucoma, early through to late progression, functional and morphological changes appear in the microvasculature of both the retina and ONH, independently of IOP (Newman et al., 2018). Ocular blood flow measurements have improved over recent years but remain technically challenging. Compared with healthy controls, ocular blood flow is disturbed in glaucoma patients and is a recognized factor that contributes to progressive visual field loss (Galassi et al., 2003; Grieshaber and Flammer, 2005). Ocular blood flow is more reduced in patients who have IOP in a normotensive range, compared with patients who experience ocular hypertension (Kaiser et al., 1997). Ocular hypotensive medications have the potential to improve ocular blood flow in the eye, but studies to date have had difficulties disentangling the effects of lowered IOP and improved circulation in the eye (Januleviciene et al., 2012).

In addition to blood flow, advances in fundus imaging have enabled vessel diameters close to the optic disc to be routinely performed on patients. Retinal microvascular caliber is therefore one of the most commonly reported biomarkers, with high reproducibility using semi-automated quantification methods (Li et al., 2013; Newman et al., 2018). However, there are limitations in the quantification of more specific vessel artifacts that require assessment by trained observers, such as focal arteriolar narrowing (Wong, 2004). Vessel caliber measurements indicate that arteriole vessel narrowing is associated with optic nerve damage and severity of optic neuropathy (Jonas and Naumann, 1989; Jonas et al., 1989; Lee et al., 1998; Papastathopoulos and Jonas, 1999). A longitudinal study of glaucoma patients linked early blood vessel narrowing with disease progression; over a 10-year follow-up period, patients with narrowed retinal arteriole caliber were associated with a greater risk of developing glaucoma (Kawasaki et al., 2013). In this particular study, RNFL measurements were not carried out, so a correlation between microvascular changes and RNFL thickness could not be determined. Nonetheless, generalized narrowing of retinal vessel caliber is a phenomenon associated with glaucomatous optic neuropathy and RNFL thinning that occurs independently of elevated IOP; ocular hypertensive patients without glaucoma pathology did not exhibit vessel narrowing (Rankin and Drance, 1996; Mitchell et al., 2005; Amerasinghe et al., 2008). This finding is corroborated in the pediatric population (De Haseth et al., 2007), lending support to the notion that vascular changes in glaucoma are independent of IOP and may be associated with other pathological features.

In addition to vessel narrowing, further along in disease progression, OCT-angiography in glaucoma patients shows reduced vessel density in retinal capillary layers (Yip et al., 2019; see also Quigley et al., 1984). In the very early stages of glaucoma, macula vessel dropout is common and there is a significant association between ONH vessel density with peripapillary RNFL thickness (Suh et al., 2016; Yarmohammadi et al., 2016a, b; Hou et al., 2019). Elevated IOP combined with decreased perfusion pressure is correlated with reduced retinal vessel density, which may lead to a reduction in blood flow to retinal tissues (Baek et al., 2019). These changes observed in humans also reflect in animal models of the disease. In a rat model of elevated IOP, there is reduced capillary volume, perimeter, diameter and density in the optic nerve head (Moreno et al., 2014). In the DBA/2J mouse model of glaucoma, choroid and retinal blood flow reduce as age and IOP increase (Lavery et al., 2012).

Of particular importance to the health of RGC axons is the microvascular perfusion at the ONH. In glaucoma there is a general compromise of the vasculature in the ONH and surrounding regions (Liu and Neufeld, 2000; Jia et al., 2012; Liu et al., 2015; Scripsema et al., 2016; Akil et al., 2017; Nascimento et al., 2019). Thus glaucoma is often associated with an unmet need for metabolites and O_2_ due to insufficient blood flow, or ‘ischemia’ (Osborne et al., 2004; Kaur et al., 2008; Schmidt et al., 2008). In fact, the posterior lamina is implicated as the primary site of disruption in glaucoma, and emerging studies show a significant decrease in vessel density and blood flow in POAG in the deeper layers of the ONH compared with controls (Nascimento et al., 2019). Microvascular density correlates with RGC axon volume across all areas at the ONH, but the correlation is greater at the posterior lamina cribrosa, further emphasizing the importance of changes in vascular parameters at this site (Kang et al., 2018). Narrowing of retinal blood vessels is also characteristic of advanced glaucomatous optic nerve damage, indicating that vascular changes occur in the retina in addition to the site of injury at the ONH (Jonas et al., 1989; Rankin and Drance, 1996). ONH blood flow velocity is reduced to a greater extent in glaucoma patients with visual field progression compared to those with non-progression (Yamazaki and Drance, 1997). Furthermore, eyes with progressive visual field defects in NTG patients had lower blood vessel velocities (Kaiser et al., 1997; Yamazaki and Drance, 1997).

Changes in vascular morphology, i.e., narrowing of vessels and complete vessel dropout, are indicative of deleterious changes in blood vessel tone and blood flow regulation at the level of the neurovascular unit (NVU). There is a higher incidence of these changes in glaucoma patients at all stages of disease progression. Interestingly, focal arterial narrowing and other microvascular changes are also associated with other non-glaucomatous optic neuropathies (Jonas et al., 1991; Rader et al., 1994), suggesting that changes in vessels occur across a wide range of IOP values and may well arise from dysfunctional RGCs, leading to impaired blood flow and vessel narrowing. When RGCs are dysfunctional, for example, RGCs that are experiencing higher levels of ROS, or cells that are undergoing cell death, they are not as metabolically active and due to lower nutritional demand, blood flow decreases. Therefore blood flow dysregulation in glaucoma likely exacerbates the progressive loss of RGCs. The next sections of this review will highlight critical pathways in blood flow regulation in the retina and ONH.

## Blood Flow Regulation in the Eye

**FIGURE 2:**
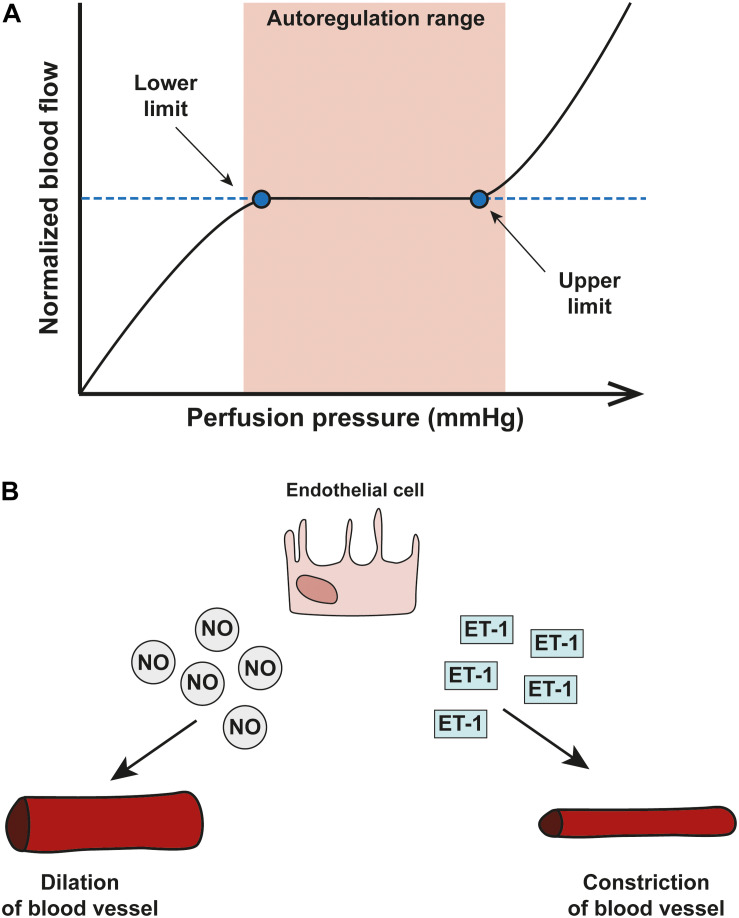
Blood flow autoregulation in the eye. **(A)** A schematic showing an autoregulation curve that describes the relationship between normalized blood flow (*y*-axis) and perfusion pressure (*x*-axis). Autoregulation can only operate within a critical range of OPP and once OPP surpasses the optimal range (shown in pink), autoregulatory systems start to break down. **(B)** The two important vasoactive substances released by endothelial cells are nitric oxide (NO) and endothelin-1 (ET-1) and autoregulation of the vascular system in the eye relies on a delicate balance between the two; NO is a potent vasodilator released by smooth muscle cells and endothelial cells which acts via pericytes to dilate capillaries. Opposite in function is ET-1, a potent vasoconstrictor.

The vessels of the retina and ONH have evolved mechanisms that enable blood flow to meet the dynamic metabolic demands of the tissue (Kur et al., 2012). Such mechanisms include tight autoregulation and neurovascular coupling. Autoregulation enables vascular beds in the retina and ONH of healthy eyes to maintain a continuous blood flow and supply of metabolites despite fluctuations in ocular perfusion pressure (OPP, Alm and Bill, 1973). To determine autoregulation capacity in patients, measurements of blood flow differences are carried out before and after the OPP is artificially increased or decreased. The normalized blood flow change represents the autoregulation capacity at any given OPP level tested. Changes in blood flow in response to OPP changes plotted on a graph constitute a classic autoregulation curve (Figure 2A). The curve includes a plateau region across a range of OPP where the blood flow is fully compensated by autoregulatory mechanisms. When the OPP fluctuations exceed the autoregulation range defined by this plateau, vasomotor adjustments are incomplete and blood flow will gradually decrease or increase passively as OPP changes. Autoregulation is achieved through changes in blood vessel tone and through neurovascular interaction. Typically, when blood vessels experience changes in blood pressure, they alter the resistance and the tone of their vessel walls as part of the ‘myogenic response’ in order to maintain a continuous flow through the tissue (Hayreh, 2001). Arterioles will contract or relax in response to an increase or decrease in intravascular pressure, respectively (Boltz et al., 2013; Prada et al., 2016).

Two key vasoactive molecules mediate blood vessel tone and blood flow: nitric oxide (NO) and endothelin-1 (ET-1; Nyborg and Nielsen, 1990). NO is a potent vasodilator released by smooth muscle cells and endothelial cells which acts via pericytes to dilate capillaries (Hayreh, 1997). ET-1 is a potent vasoconstrictor released from endothelial cells that acts on three primary receptors: ET_A_, ET_B1_, and ET_B2_ receptors. ET_A_ receptors are present in vascular smooth muscle and mediate the vasoconstrictive properties of ET-1 (Orgul et al., 1999; Resch et al., 2009a; Schmidl et al., 2011). ET_B1_ receptors are present on endothelial cells and facilitate vasodilation (Schmidl et al., 2011). ET_B2_ receptors on the other hand mediate constriction of vessels (Pollock et al., 1995).

Aside from its role in regulating blood flow, ET-1 mediates a multitude of responses in the retina through targeting ET-1 receptors on several cell types. In rodents, intravitreal and peribulbar administration of ET-1 activates receptors on RGCs altering the rate of RGC axonal transport and promotes apoptotic cell death of RGCs (Stokely et al., 2002; Yorio et al., 2002; Chauhan et al., 2004; Lau et al., 2006; Taniguchi et al., 2006; Krishnamoorthy et al., 2008). ET-1 receptor activation on astrocytes promotes their proliferation (Lau et al., 2006). Moreover, ET-1 reduces expression of RGC mitochondrial oxidase enzymes, implicating a role for ET-1 in RGC bioenergetics (Chaphalkar et al., 2020). The effect of ET-1 on RGCs and other cell types directly is beyond the scope of this review, but reviewed well in Shoshani et al. (2012).

In vascular autoregulation, a delicate balance between concentrations of NO and ET-1 mediates appropriate vessel response to maintain blood flow (Figure 2B; Orgul et al., 1999; Venkataraman et al., 2010). In glaucoma, mounting evidence suggests dysfunction in the pathways of vasoactive mediators.

## Blood Flow Regulation Is Impaired in Glaucoma

Patients with glaucomatous optic neuropathy have abnormal autoregulatory responses due to the dysfunction of the cells involved in these processes (Pournaras et al., 2004; Galambos et al., 2006; Feke and Pasquale, 2008; Prada et al., 2016). Cellular stress derived from pressure changes at the ONH, combined with impaired autoregulatory responses which triggering ischemia may accelerate glaucomatous RGC degeneration (Trible et al., 1993).

Elevated serum levels of ET-1 and other biochemical markers of endothelial function in the aqueous humor of glaucoma patients suggest that endothelial dysfunction is associated with disease pathology (Sugiyama et al., 1995; Noske et al., 1997; Resch et al., 2009a; Ghanem et al., 2011; Cellini et al., 2012; Li et al., 2016). There is a statistically significant correlation between microvascular endothelial function and severity of POAG in the Malay population (Bukhari et al., 2016). In patients, blocking both ET_A_ and ET_B_ receptors results in increased blood flow through the retina, choroid and ONH. In DBA/2J mouse studies, delivery of bodentan, a dual ET_A_ and ET_B_ receptor blocker, significantly protects against glaucomatous damage at the ONH (Resch et al., 2009b; Howell et al., 2011). In addition, administration of ET-1 in proximity to the optic nerve head leads to ischemia, and the appearance of clinical indications of glaucoma including increased cupping of the optic disc, which leads to subsequent RGC loss (Orgul et al., 1996; Chauhan et al., 2004; Cioffi et al., 2004). Mice with endothelium-specific overexpression of ET-1 exhibit both retinal vascular dysfunction and progressive loss of RGCs over 10–12 months (Mi et al., 2012). Importantly, a recent study in mice directly links IOP elevation to vascular endothelial dysfunction, which bolsters findings of endothelial dysfunction in glaucoma patients where elevated IOP is apparent. In the study, elevated IOP blunts retinal arteriole reactivity in response to the endothelium-dependent vasodilator acetylcholine, but not to the endothelium-independent nitric oxide donor, nitroprusside. Also, retinal arteriole responses to changes in perfusion pressure are compromised in eyes with elevated pressure, suggesting that autoregulation is impaired (Gericke et al., 2019). In the DBA/2J mouse model of inherited glaucoma, several molecular changes in the ONH are detectable before damage to optic nerve axons have been elucidated, and these include endothelin induction in microglia (Howell et al., 2011).

As well as perturbations in the endothelin-1 pathway, there is also longstanding evidence that impaired NO signaling is implicated in glaucoma (Haefliger and Anderson, 1997; Polak et al., 2007; Wareham et al., 2018). NO is a gaseous signaling molecule, however, high NO concentrations can be neurotoxic and induce oxidative stress through the formation of reactive nitrogen species (Pacher et al., 2007). In a rat model of glaucoma, RGC degeneration is linked with increased nNOS expression and RGC loss was prevented by NOS inhibition (Neufeld et al., 2002). NO is not always deleterious to ocular function, and a delicate balance in NO production is therefore necessary to support a healthy cellular environment. Production of NO by NO-synthase (NOS) enzymes in the ONH is essential for controlling the vascular tone of the region (Haefliger et al., 1992, 1993, 1999). When NO production is blocked systemically by inhibition of NOS in glaucoma patients, both choroidal and ONH blood flow do not decrease to the same extent as in healthy patient controls, suggesting that elevated basal NO in glaucoma patients may be a compensatory mechanism to ensure optimal ocular blood flow (Polak et al., 2007). On the other hand reports of decreased levels of NO were found in the aqueous humor of POAG patients (Doganay et al., 2002), as well as a reduction in the levels of cGMP, a signaling molecule downstream of NO production (Galassi et al., 2004). In animals, impaired NO signaling has also been linked with glaucomatous characteristics. A mouse line deficient in the alpha subunit of the guanylate cyclase (GC1^–/–^), an enzyme activated by NO and responsible for the production of cGMP), develop POAG over time, characterized by RGC axon loss, modest increases in IOP and impaired retinal vascular function (Buys et al., 2013). RGC loss is linked to deficiencies in the NO-cGMP signaling pathway in two animal models of glaucoma and treatment with tadalafil, a phosphodiesterase inhibitor prevents RGC degeneration, independently of IOP (Wareham et al., 2018).

Dysfunction in the NO signaling pathway, either through up-regulation, or down-regulation, is a likely contributor to abnormal ocular blood flow; an increase or decrease in NO shifts the balance between vasoconstrictive and vasodilatory mediators. Poor ocular perfusion is directly detrimental to RGC health, leading to ischemia, oxidative stress, and lack of metabolic support. In addition, a reduction in ocular perfusion may also increase the sensitivity of the cells to other glaucoma-related stressors conveyed at the ONH that further exacerbate disease progression.

## Neurovascular Coupling in Glaucoma

**FIGURE 3:**
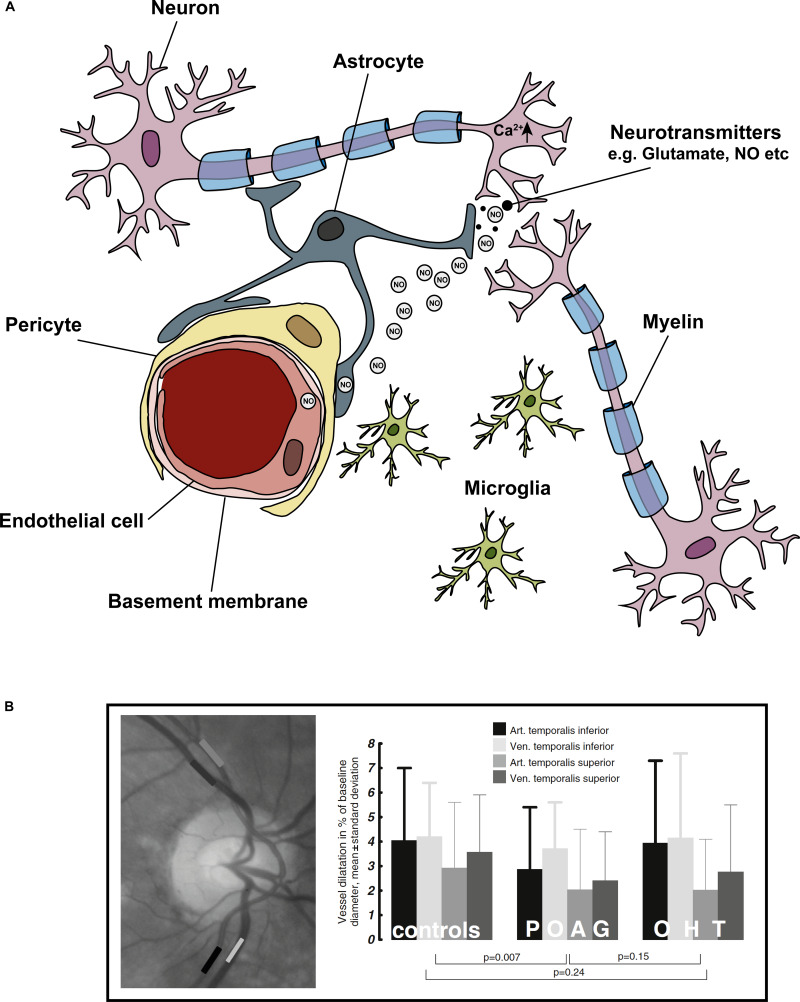
Cells of the ‘neurovascular unit’ and the light flicker response. Neurovascular coupling describes the coupling of neuronal activity to vascular responses. **(A)** Shows the cells comprising the NVU, these include neurons (in the eye specifically – RGCs), astrocytes, microglia, pericytes, and endothelial cells. In general, a spike in neuronal activity leads to an increase in intracellular Ca^2+^, which generates NO. NO diffuses to local blood vessel endothelial cells, activating K^+^ channels, which leads to downstream vasodilation and increased blood flow. The light flicker response demonstrates the tight coupling of neuronal activity (in response to light) and change in vessel diameters in the retina. In glaucoma, this light flicker response is diminished. **(B)** To measure the light flicker response in the retina, a fundus video is used (image left) where the temporal inferior artery and vein, and temporal superior artery and vein are clearly visible. Areas of analysis are shown in grayscale boxes. Graphical representation the light flicker response in control, ocular hypertension (OHT) and glaucoma patients shows diminished vessel response with disease. Figure adapted from Gugleta et al. (2013b).

Neuronal activity and blood flow are tightly coupled in the central nervous system in a phenomenon known as ‘functional hyperemia’ – a spike in neuronal activity evokes increased blood flow to the area (Roy and Sherrington, 1890). After initial observations in the brain, the general consensus was that homeostatic regulation of blood flow was dependent on local metabolite concentration in a negative feedback loop. In this mechanism, increased neuronal activity leads to increased energy demand, for example, the additional ATP consumption that is required to reset ion gradients after an action potential (Attwell et al., 2010). A reduction in ATP is perceived as an increased need for metabolites in tissues and thus induces dilation of blood vessels. However, the vascular supply to tissues after neuronal activity far supersedes the metabolic requirements of the tissue, so a feedback mechanism working alone has been discredited (Powers et al., 1996). More recent work has shown that glial cells play a major role in neurovascular coupling (NVC) via a feedforward mechanism (Vaucher et al., 1997; Attwell et al., 2010; Petzold and Murthy, 2011). In this process, neuronal activity leads to neuronal signaling to nearby blood vessels or astrocytes, which leads to the release of vasoactive agents thereby increasing blood supply (Attwell et al., 2010). The latest consensus is that both feedforward and feedback mechanisms are at play; the initial feed-forward mechanisms that over-supply neurons with nutrients may be balanced by a feedback mechanism that is metabolism-dependent and responsive to the accumulation of vasoactive metabolic by-products (Iadecola, 2017). The objective of these mechanisms medicated by the NVU is to meet the metabolic needs of the neurons. A triumvirate of cell types comprise the NVU (Figure 3A): vascular cells (vascular smooth muscle cells, pericytes and endothelial cells), glial cells (astrocytes, microglia, and oligodendrocytes), and neurons (Iadecola, 2004; Attwell et al., 2010; Hamilton et al., 2010; Winkler et al., 2011).

The NVC response has been elegantly demonstrated in the ONH and retina with experiments investigating hemodynamic responses to flicker-light stimulation (Riva et al., 1986, 1997; Garhofer et al., 2003, 2005; Gugleta et al., 2012, 2013a). In glaucoma patients, this response is dysfunctional; flicker-light induced retinal vasodilation responses are diminished (Figure 3B, Garhofer et al., 2004). This impaired response is likely due to reduced neuronal activity and altered glial cell function in the disease (Kornzweig et al., 1968; Hernandez et al., 2008). However, it could also be directly related to fluctuations in IOP, or vascular dysfunction in the tissue. In an experiment where short term IOP elevations of up to 43 mmHg were inflicted in healthy subjects, flicker-light stimulations in the retina were maintained, suggesting that diminished responses in glaucoma patients are not necessarily directly due to changes in IOP alone (Garhofer et al., 2005). Thus, the dysfunctional NVC response observed in glaucoma patients probably arises from dysfunction at the cellular level provoked by other, non-IOP-related stressors.

## Pericytes and the NVU

The involvement of pericytes in the regulation of blood flow in the retina and the relation to glaucomatous disease has been largely unexplored. Pericytes are embedded in the basement membranes of microvessels, and extend their processes along capillaries, pre-capillary arterioles and post-capillary venules (Sweeney et al., 2016). Pericytes express several types of muscle contractile proteins (Herman and D’amore, 1985) and are involved in propagating vasomotor signals along the length of capillaries (Peppiatt et al., 2006; Puro, 2007). Pericytes are responsive to vasoactive molecules described earlier, e.g., NO, and other circulating metabolites, such as ATP (Haefliger and Anderson, 1997; Kawamura et al., 2003). Pericytes function similarly to smooth muscle and endothelial cells, possessing a number of ion channels and transporters that help to mediate changes in capillary diameter. Like smooth muscle, alterations in pericyte tone and contractile ability change with intracellular Ca^2+^ levels (Sakagami et al., 1999, 2001; Wu et al., 2003). The overall tone of pericyte-containing microvessels is the result of a balance between Ca^2+^-mediated contractility and by NO-mediated relaxation (Kutcher et al., 2007).

As an integral cell of the NVU, it is unlikely that pericytes are immune to cellular changes or dysregulation. In an experiment mimicking glaucoma in mice, elevated IOP stimulated the expression of β-III-tubulin, a neuronal cell marker, in both pericytes and endothelial cells, suggesting that vascular cells respond to changes in IOP via alterations in protein expression (Prokosch et al., 2019). It is not yet understood how these expression changes relate to regulation of blood flow or in NVC responses in the retina, however, the results suggest that pericytes respond to IOP-related stress through changes in gene expression. Furthermore, pericytes themselves are vulnerable in ischemic conditions; retinal ischemia reduces the ability of pericytes to relax after constriction, leading to a further decrease in blood flow (Hall et al., 2014; Sweeney et al., 2016; Alarcon-Martinez et al., 2019). Glaucoma has been described as a vasospastic disease, whereby retinal ischemia-reperfusion injury repeatedly occurs, rather than a single ischemic event (Flammer et al., 2001). Reduced retinal blood flow in glaucoma may lead to pericyte dysfunction, which may further impact reperfusion of retinal tissue. Glaucoma is associated with aging, and changes in pericytes that occur with age may also contribute to, and encourage, degeneration of RGCs. In the aged rat retina, there is breakdown of the normal vascular architecture and reduced pericyte-endothelial cell contact (Hughes et al., 2006). Pericytes express a number of gap junction proteins that facilitate cell–cell communication with other cells of the NVU, and expression changes of these gap junctions in glaucoma may also play a role in vascular dysfunction (discussed below). With their central role in retinal perfusion, the role of pericytes in ocular perfusion and blood flow in glaucoma is crucial in our understanding of vascular dysfunction in the disease and is an area that warrants further investigation.

## Vascular Connectivity in Glaucoma

Astrocytes are the predominant glial cell type of the unmyelinated ONH, and their close proximity to blood vessels hints at their importance in mediating blood flow (Balaratnasingam et al., 2014). Astrocytes react to neurotransmitters released during neuronal activity (such as glutamate) by increasing their intracellular Ca^2+^ levels, prompting the release of vasoactive substances, such as K^+^ (Porter and Mccarthy, 1996; Filosa et al., 2006). Astrocytes are key players in the neurovascular coupling response and in healthy eyes, they are quiescent and mediate normal neurovascular responses (Bachoo et al., 2004), however, in response to changes in the ONH environment, such as changes in IOP, or ischemia, astrocytes become reactive, promoting the degradation of RGCs and their axons (Varela and Hernandez, 1997; Hernandez et al., 2008). Since changes in glial reactivity are significantly implicated in various stages of glaucoma, it is plausible that all cells of the NVU in some way have a role to play in the breakdown of NVC in the retina and ONH that contributes to disease progression. Multiple pathways integral to the NVU are differentially regulated in glaucoma, and evidence for their role in glaucoma progression is outlined below.

In NVC, NO surfaces as an important player when considering direct neuronal signaling to blood vessels. An increase in neuronal activity causes the synaptic release of glutamate, activating NMDA receptors on neurons, leading to increased intracellular levels of Ca^2+^. An elevated level of Ca^2+^ triggers a cascade of events leading to the activation of nNOS, which generates intracellular NO. NO can directly activate BKCa channels (Bolotina et al., 1994), or indirect activation can occur through NO-derived cGMP (Stumpff et al., 1997). Activation of BKCa channels leads to K^+^ efflux and cell hyperpolarization. Cell hyperpolarization causes voltage-operated (L-type) Ca^2+^ channels to close, reducing Ca^2+^ influx, leading to vasorelaxation of vascular smooth muscle cells. This chain of events leads to vasodilation and increased blood flow (Cavet et al., 2014; Prada et al., 2016). Dysfunctional NO signaling is implicated in glaucoma pathogenesis and endothelial dysfunction. Inhibitors of NOS attenuate light-induced vasodilation in the retina and the ONH (Kondo et al., 1997) and increased levels of NO are observed in the ONH in response to changes in neuronal activity in flicker-stimulation (Buerk and Riva, 2002). Reactive astrocytes have been shown to produce excess amounts of NO through the activation of inducible-NOS (iNOS) (Neufeld et al., 1997; Liu and Neufeld, 2000), increasing free radicals and causing damage to local axons. Interestingly, despite high levels of NO in the ONH, blood flow is still often impaired. NO signaling represents a double-edged sword paradigm; NO in excess is detrimental to cells, causing great damage to cellular components and their dysfunction; this likely impacts vascular function. However, lower levels of NO, however, can fine-tune vascular responses. The role of NO in the NVC response may therefore lie in modulation, rather than mediation of the response; in the rat retina, high concentrations of NO leads to vasodilation of nearby blood vessels, whereas lower concentrations causes vasoconstriction (Metea and Newman, 2006). Impaired NO signaling in astrocytes may indeed contribute to atypical neurovascular responses, causing reductions in ocular blood flow that ultimately lead to ischemia and subsequent RGC degeneration.

In the retina and optic nerve, gap junction channels between cells mediate intercellular communication, such as the communication between astrocytes and blood vessels. This communication can occur from glial cell to glial cell, or through glial cells communication to other cell types. The concept of neuron-glial connectivity, or ‘gliotransmission,’ was initially introduced to account for the active transfer of neuroactive molecules, from glia to neurons (Bezzi and Volterra, 2001). Initial evidence that connexin channels played a role in neuron-glial interactions was demonstrated using co-culture models and *ex vivo* brain slices (Nedergaard, 1994; Froes et al., 1999; Giaume and Theis, 2010). Increases in Ca^2+^ generated in astrocytes triggered Ca^2+^ responses in co-cultured neurons; an effect abolished by connexin channel blockers, suggesting that astrocytic-neuronal connectivity is mediated, in part by gap junctions. These junctions are integral to cell-to-cell transfer of electrical conductance (Na^+^, K^+^, and Ca^2+^) and small molecules such as ATP/ADP, glutamate, and glucose, and second messengers (e.g., NO, cGMP, and cAMP; Bloomfield and Volgyi, 2009; Giaume et al., 2020). The five major neuronal classes in the vertebrate retina form diverse coupling networks by gap junctions formed by connexin proteins (Sohl and Willecke, 2003; Sohl et al., 2005). Gap junction channels have been implicated in numerous cellular processes including in maintaining ionic balance, synaptic plasticity, metabolic substrate trafficking, and cellular survival (Andrade-Rozental et al., 2000; Wright et al., 2001; Bloomfield and Volgyi, 2009; Spray et al., 2013). Gap junction-mediated gliotransmission is a vastly growing field, and we refer the reader to a recent review (Giaume et al., 2020).

**FIGURE 4:**
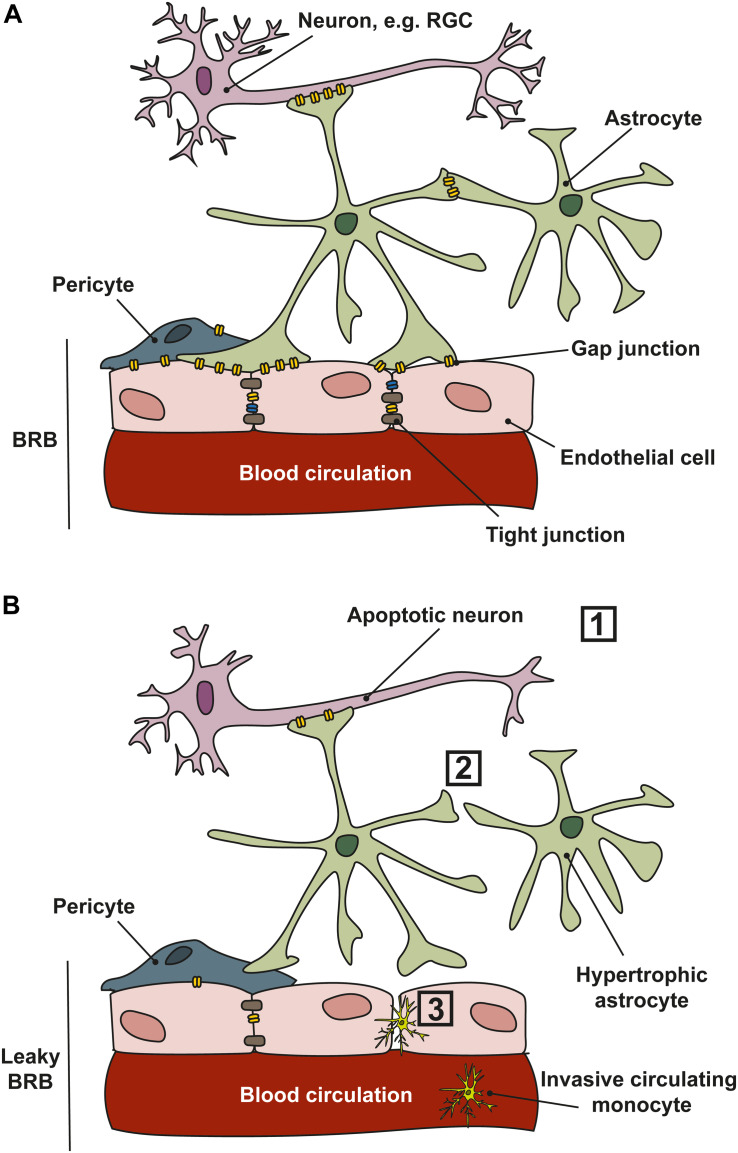
Cell communication in the NVU. **(A)** In healthy tissues, retinal ganglion cell (RGC) neurons, astrocytes and endothelial cells communicate through gap junctions which permit the movement of electrical conductance (Na^+^, K^+^, and Ca^2+^) and small molecules such as ATP/ADP, glutamate, and glucose, and second messengers. Gap junctions exist in the retina and ONH between neurons, glia and between vascular cells. **(B)** In glaucoma, several changes occur that affect communication between the cells of the NVU. (1) RGCs are vulnerable to stressors that lead to apoptosis; (2) cell–cell communication is lost as astrocytes and other glial cells become reactive and gap junction expression is reduced; (3) a reduction in gap junction expression, and loss of tight junctions between vascular cells leads to a leaky blood–retinal-barrier (BRB), allowing the infiltration of circulating monocytes into the retina and ONH.

In the retinal NVU, gap junctions are integral in the maintenance of the blood–retinal-barrier (Figure 4), and in glaucoma, changes in gap junction expression may compromise the blood–retinal-barrier, exacerbating neuronal degeneration (Figure 4B). Ischemia and ocular perfusion instability in glaucoma damages astrocyte–astrocyte gap junctions; under experimental elevated-IOP conditions, there is decreased gap junction communication between ONH astrocytes (Malone et al., 2007). This may lead to loss of continuity and communication between astrocytes and other cells of the NVU, including pericytes and neurons, which can disrupt ionic and metabolic homeostasis in the tissue and eventually alter blood flow (Hernandez et al., 2008). Indeed, expression of Cx43 in pericytes is important in the development, maturation, and maintenance of the blood–brain-barrier and also in retinal blood flow (Trost et al., 2016; Giaume et al., 2020), but changes in connexin expression in pericytes as they relate to glaucoma progression have not been investigated to date. In healthy rabbits, uncoupling the gap junctions between astrocytes impairs ONH blood flow regulation (Shibata et al., 2011). Ischemia also differentially regulates the hemichannels of gap junctions (Thompson et al., 2006). In human glaucomatous eyes, Cx43 expression is upregulated at the level of the lamina cribrosa and in the peripapillary and mid-peripheral retina in association with glial activation (Kerr et al., 2012). *In vitro* studies show that an increase in hydrostatic pressure leads to loss of gap junction communication and redistribution in human astrocytes (Malone et al., 2007). Conversely, in other models of retinal ischemia, blockade of Cx43 reduced overall cell death and injury in the retina and ONH (Kerr et al., 2012).

In the optic nerve and ONH, the processes of astrocytes are interconnected via gap junction proteins Cx30 and Cx43, allowing intercellular communication that contributes to maintaining a homeostatic cellular environment (Quigley, 1977; Rose and Ransom, 1997; Rash et al., 2001). Expression of Cx43 on astrocytes increases during chronic stress (Frisch et al., 2003; Giaume and Theis, 2010; Kerr et al., 2011). *In vitro*, elevated hydrostatic pressure causes astrocytes to alter localization and phosphorylation state of Cx43 (Malone et al., 2007). Increased phosphorylation of Cx43 leads to gap junction uncoupling (Warn-Cramer et al., 1998), whereas decreased phosphorylation is correlated with a decrease in gap junction communication (Godwin et al., 1993) or an increased in gap junction conductance (Moreno et al., 1994). These studies do not provide direct *in vivo* evidence for increased connectivity between astrocytes under glaucoma stress but suggest that glaucoma-related stresses can alter the connectivity of astrocyte gap junction proteins and their activity.

The role of gap junctions in the vasculature of the retina and ONH is less well established. Direct electrical communication of vascular cells via gap junctions has been shown to mediate the vasomotor tone and propagation of vasomotor response in the retina (Ivanova et al., 2017). Most recently, expression of another gap junction, Cx45, was shown to form electrical synapses on RGC axons in the optic nerve (Smedowski et al., 2020). In other degenerative eye diseases, such as diabetic retinopathy, down-regulation of gap junctions leads to the breakdown of the blood–retinal-barrier (Oku et al., 2001; Bobbie et al., 2010; Tien et al., 2016). There is evidence elsewhere in the CNS for gap junction association with tight junctions; in the porcine blood–brain-barrier, Cx43- and Cx40-containing gap junctions are required for the endothelial barrier (Nagasawa et al., 2006). In mice, connexins Cx43 and Cx40 are expressed throughout the retina on glia and retinal vasculature, whilst Cx37 is expressed along endothelial cells throughout the retinal vascular tree (Ivanova et al., 2019). In particular, Cx43 is expressed at tight junctions and between astroglia and endothelial cells, suggesting that these gap junctions have an integral role in maintenance of the blood–retinal-barrier (Ivanova et al., 2019). Another gap junction, not yet linked to vascular communication in the retina, is Cx36 which is found throughout the inner retina, but not the optic nerve. The expression of Cx36 been shown to increase with elevated IOP in a mouse model of glaucoma (Akopian et al., 2017). Blockade of Cx36 prevents RGC degeneration, suggesting a role of Cx36 in promoting apoptosis through inter-neuronal communication of death-signals, however, a role for Cx36 in the function of the blood–retinal-barrier is yet to be explored.

## Conclusion and Future Directions

The observation that systemic and ocular vascular dysfunctions are correlated with the incidence of glaucoma raises an important question; does vascular dysfunction precede glaucomatous optic neuropathy, increasing the sensitivity of RGCs to pressure at the ONH, or is it merely a secondary consequence of other pathological changes in the disease, e.g., increased inflammation? Treatments so far have focused on the anterior chamber with current therapies aimed at lowering IOP, the only modifiable risk factor for the disease. Such drugs address IOP by modulating the amount of aqueous humor produced by the ciliary body, or by improving outflow through the trabecular meshwork (Weinreb et al., 2014). These treatments have variable success rates, with many patients requiring additional invasive surgery. A good proportion of patients continue to progress despite adhering to these treatment regimens – visual field loss is inevitable. In most cases treatments that target IOP serve only to delay progression of the disease, they do not prevent degeneration of RGCs and their axons.

The fact that glaucomatous optic neuropathy occurs at all levels of IOP, and that patients progress regardless of interventions to regulate IOP suggests that there are other factors that contribute to the degeneration of RGCs and their axons in the visual projection. As we have outlined, these factors include increased vascular dysfunction, and ocular hemodynamics are critical players in the progression of glaucoma (Flammer, 1994). Systemic vascular diseases such as hypertension and hypotension are correlated with glaucoma (Tielsch et al., 1995), and changes in the vasculature of glaucoma patients, for example, disc hemorrhages are also evident in both early and late stages of the disease. Ischemia at the ONH leads to increases in oxidative stress and inflammation, as well as a decrease in the supply of essential nutrients and metabolites. Such stressors not only lead to degeneration of RGCs directly, but also render RGCs more sensitive to mechanical stresses conveyed at the ONH. An important question is how all of these glaucomatous stressors interplay to cause sensitivity of RGCs. A novel hypothesis that may marry together vascular dysfunction with inflammation and biomechanical stress of tissue in the retina and ONH is the idea that the eye possesses an ocular glymphatic system (Wostyn et al., 2017). Such a glymphatic system would have a similar role in the eye as it does in the brain, primarily as an exit for toxic waste products. It would be interesting to investigate whether there is paravascular communication between the surroundings of the retinal vascular system and the surroundings of the central retinal vessels in the optic nerve, and how vascular factors may alter glymphatic flow.

As technologies for *in vivo* imaging of vasculature in the retina and ONH of glaucoma patients improve, evidence is mounting in support of vascular abnormalities coinciding with optic neuropathy in glaucoma. A key question moving forward in glaucoma research is how can we target vascular function in the design and development of new treatments? Vascular dysfunction in glaucoma likely arises from impaired functioning of cells in the NVU, and a loss of connectivity between neurons, glia, and endothelial cells. Thus, future research into the pathways involved in this intercellular communication, as outlined in this review, is key to our understanding of the role of NVC in glaucomatous disease and efforts to delineate the temporal changes in NVC and RGC death require urgent investigation. Vascular dysfunction occurs at all levels of IOP and can affect RGC health directly, however, we do not fully understand the role of these pathways in RGC survival. Further work in these areas will lead to therapies that are aimed at mediating proper vascular regulation and therapies that promote neurovascular interactions. Both of these are attractive novel areas to explore in the search for neuroprotective therapies in glaucoma.

## Author Contributions

LW and DC collected the analysis and wrote the manuscript.

## Conflict of Interest

The authors declare that the research was conducted in the absence of any commercial or financial relationships that could be construed as a potential conflict of interest.
